# Inguinal Trusses

**Published:** 1904-05-07

**Authors:** 


### INGUINAL TRUSSES.

It must have happened to most general practitioners at the commencement of their careers, if not later on, to find great difficulty in obtaining for their patients trusses which combine to a satisfactory degree comfort with efficiency. Some useful practical hints on the subject are given by Mr. W. McAdam Eccles.[1] He prefaces his remarks with the opinion that for most forms of hernia the best variety of truss is one which conforms to a type having a pad to cover the aperture, placed upon a spring which encircles the trunk, and with accessory straps for better support. Continuing, Mr. Eccles lays down the following as the essentials of a well-made ordinary inguinal truss :—(1) The pad has its foundation of soft iron. This should be oval in shape, with two studs on its anterior surface, and two sharp points on its posterior aspect. The size of an inguinal pad for an adult is about 4 inches long, 2 inches broad, and $1\frac{1}{2}$ inches thick when its facing of cork is in position. The lower border of the pad, which is curved, should project only half an inch below the inferior edge of the spring. It must not descend lower than this, or there will be a tendency for the pad to become displaced upwards by the movement of the thigh. The pad should be solid, and fixed immovably to the spring by rivets. (2) The spring is required to counteract the intra-abdominal pressure. The points to be considered in the spring are its length and breadth, the curve, the temper, and the free end. In a single truss the spring should be more than a semicircle—it should pass round the pelvis and reach to a point just behind the anterior superior iliac spine on the sound side. From this point the coverings of the spring should be prolonged as a cross strap, which is fastened to the upper stud on the face of the pad. As a rule the best breadth for the spring is half an inch or a little more. The curve should be such that the pad is on a plane only slightly posterior to that of the anterior portion of the spring. If the curve is too great the pressure will be excessive. The temper of the steel forming the spring must be such as to allow considerable opening out of the curve without the least liability of breaking. In order that the spring may rest flat upon the body, the lower edge of it must be the arc of a larger circle than the upper edge, owing to the increasing circumference of the pelvis, against which the spring is applied. If this rule be neglected the truss will rest only upon its lower edge, and will be exceedingly uncomfortable. This is one of the most important practical points of all, if a comfortable instrument is to be provided, and yet it is very frequently neglected by the makers of trusses. Lastly, the termination of the spring should be hammered out in the process of manufacture, so that it becomes thin and flat, and therefore much more flexible than the remainder and more likely to cling steadily to the hip. (3) Every inguinal truss should be provided with an under-strap, and the wearer should look upon it as an essential part of his instrument. The use of the strap is to prevent the spring from riding up over the hip bone, to secure the pad in position, and to give the latter a slight tilt so that it will face a little upward. The proper adjustment of the under-strap is all important. It should be firmly fixed to that part of the spring which is just behind the anterior superior spine of the ilium on the same side as the hernia. It is then carried downwards and backwards along the gluteal fold, across the perinæum, and upwards to fasten in front to the lower stud on the front of the pad. A common mistake is to fasten the under-strap to the spring at or near the middle line behind, when it causes so much discomfort and is so inefficacious that the patient is likely to discard it altogether. In conclusion, Mr. Eccles states that if a patient is provided with a truss of the proper character he will come to look upon his instrument as little more than part of his ordinary clothing, and as giving him less inconvenience even than his boots.

[1] Med. Press and Circ., March 16.

